# Coherent and Incoherent Ultrafast Dynamics in Colloidal Gold Nanorods

**DOI:** 10.1021/acs.jpclett.3c03226

**Published:** 2024-01-03

**Authors:** Federico Toffoletti, Elisabetta Collini

**Affiliations:** † Department of Chemical Sciences, University of Padova, Via Marzolo 1, 35131 Padova, Italy; ‡ Padua Quantum Technologies Research Center, Via Gradenigo 6/A, 35131 Padova, Italy

## Abstract

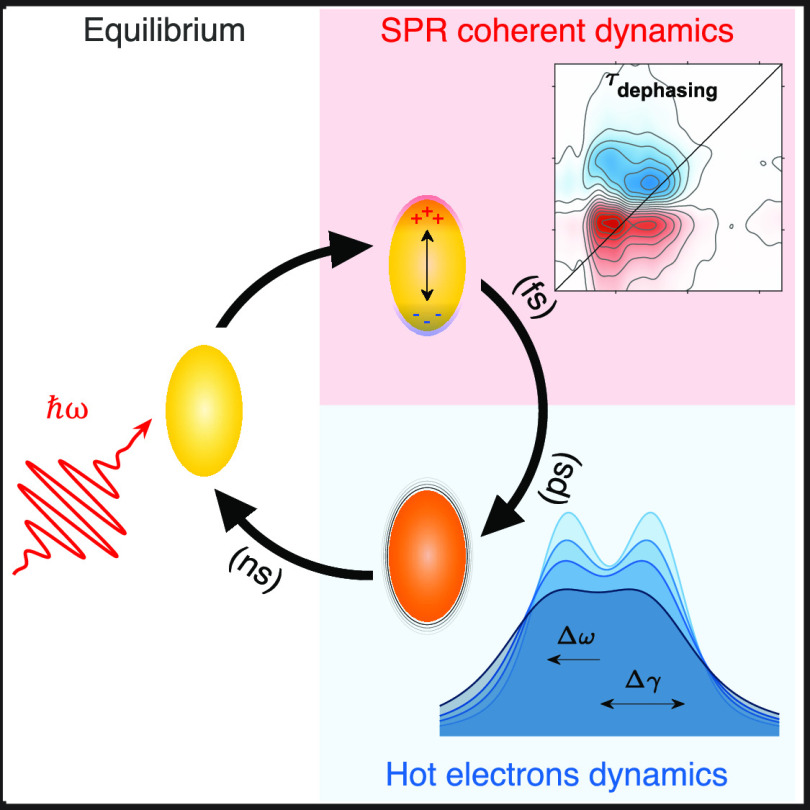

The study of the mechanisms that control the ultrafast dynamics in gold nanoparticles is gaining more attention, as these nanomaterials can be used to create nanoarchitectures with outstanding optical properties. Here pump–probe and two-dimensional electronic spectroscopy have been synergistically employed to investigate the early ultrafast femtosecond processes following photoexcitation in colloidal gold nanorods with low aspect ratio. Complementary insights into the coherent plasmonic dynamics at the femtosecond time scale and incoherent hot electron dynamics over picosecond time scales have been obtained, including important information on the different sensitivity to the pump fluence of the longitudinal and transverse plasmons and their different contributions to the photoinduced broadening and shift.

Nobel-metal plasmonic nanostructures are renowned for their significant optical nonlinear properties, which arise from the complex electron dynamics occurring within the first picoseconds following photoexcitation.^1−5^ When a metallic nanoparticle is irradiated by a resonant electromagnetic field, interband/intraband transitions or surface plasmon resonance (SPR) can be activated, depending on the frequency of the incident field. SPR consists of a collective coherent oscillation of the conduction band electrons that rapidly dephases to generate a distribution of high-energy electrons, leading to hot electron distribution through electron–electron scattering. These ultrafast dynamic processes profoundly influence the optical response. Therefore, the study of both coherent oscillation and hot electron dynamics in plasmonic nanosystems has been the subject of extensive research in the last decades due to their vast potential applications across fields such as sensing,^6−9^ phototherapy,^10^ photonics,^11^ and optoelectronics,^12^ among others.^1−5^ Numerous studies have already been conducted to establish the relationship between the dimensions, shapes, chemical nature of the nanostructures, and their optical properties.^1,4,13−17^ Nonetheless, several aspects connected with the early steps of electronic relaxation in the femtosecond regime immediately after photoexcitation remain elusive.

To contribute to filling this gap, in this work, we combine transient absorption (TA) and two-dimensional electronic spectroscopy (2DES) to comprehensively characterize the femtosecond nonlinear properties of a specific nanosystem. Our attention was focused in particular on colloidal gold nanorods (NRs) with a low aspect ratio (AR) in water suspensions. This system is particularly intriguing because it exhibits two SPRs along the longitudinal (LSPR) and transverse (TSPR) axes, close enough in energy to allow the simultaneous investigation of the photophysical behavior of the electrons involved in both resonances under the same experimental conditions. Furthermore, these nanoparticles offer an ideal platform for the preparation of nanoarchitectures fulfilling the strong light–matter coupling conditions, which are attracting increasing interest due to their peculiar optical properties.^18−22^ Therefore, gaining knowledge about the ultrafast behavior of NRs serves as an essential preliminary initial step toward a better understanding of more complex structures.^18,23^

The NRs under investigation were prepared according to the literature procedure,^24,25^ as described in the Supporting Information. Their morphological properties were investigated through transmission electron microscopy (TEM), which proved the low AR and regular ellipsoidal shape (Figure 1a). The distribution of AR obtained from the analysis of about 300 NRs is shown in Figure 1b. More details on dimensional analysis are reported in the Supporting Information. We obtained well-dispersed gold NRs with AR of 1.8 ± 0.2, length of 29 ± 4 nm, and width of 17 ± 2 nm. The AR is the most relevant parameter for the optical properties as it is well-known that the spectral position of TSPR remains basically unchanged, while LSPR is sensitive to AR and experiences progressively greater red-shifts at increasing values of AR.^4,26−29^ Differently from most of the Au NRs usually synthesized and characterized in the literature, these NRs are characterized by a very low AR, such that the LSPR and the TSPR are partially overlapped (Figure 1c). These two peaks are identified in the absorption spectrum at 2.14 and 2.37 eV, respectively.

**Figure 1:**
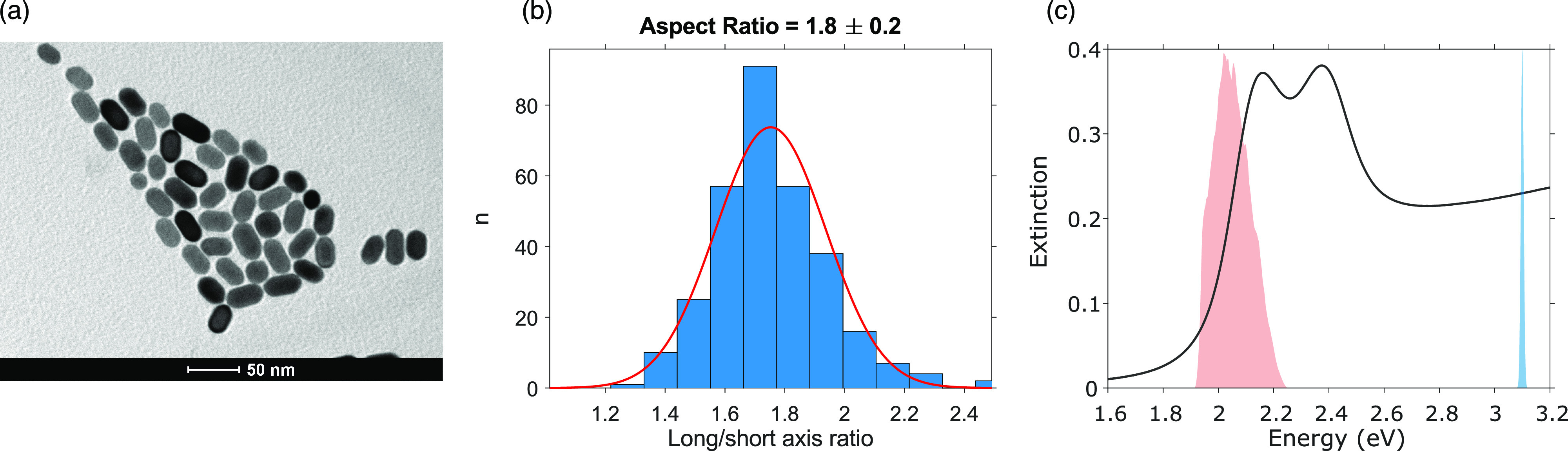
(a) TEM image of CTAB capped Au NRs dispersed in water. (b) Size distributions of AR obtained from the analysis of up to 300 NRs. (c) Extinction spectrum of NRs suspended in water (black line). The laser spectral profile used for 2DES measurements is represented as a red area, while in blue we report the excitation profile of the pump beam in transient absorption experiments.

The femtosecond dynamics of the NRs were first investigated by pump–probe spectroscopy, one of the most commonly used techniques to study the ultrafast dynamics of noble metal nanoparticles.^2,16,30−38^ In our experiments, the relaxation of the systems was investigated in the first nanosecond after photoexcitation, with a time resolution of about 150 fs. The pump pulse was centered at 3.1 eV (Figure 1c), while the probe was a white light supercontinuum (1.8–2.6 eV). Measures were conducted with different pump fluences (from about 100 to 700 μJ/cm^2^) to explore the power dependence. More details about the experimental setup can be found in the Supporting Information.

Figure 2 shows two examples of the results of the pump–probe experiments at two values of pump fluence (700 and 420 μJ/cm^2^). Each plot is a 2D map representation of the differential absorption Δ*A*(ω, *t*_d_), defined as the difference between the absorption spectrum of the sample with and without the pump pulse, as a function of probing energy (ω, eV) and pump–probe time delay (*t*_d_, ps). A TA spectrum is obtained by plotting Δ*A* as a function of the probe energy at a fixed value of delay time *t*_d_.

**Figure 2:**
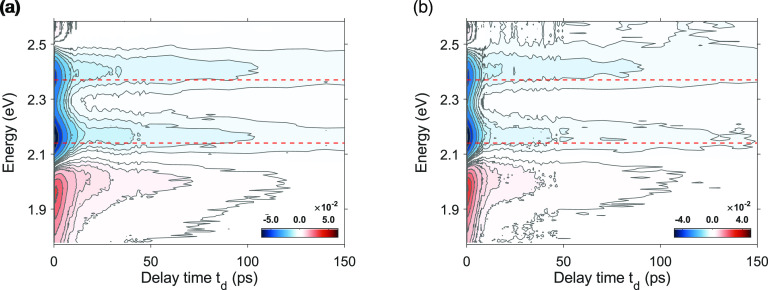
2D map representation of the differential absorption (Δ*A*, colorscale) as a function of probing energy (eV) and pump–probe time delay (ps). The data are obtained upon excitation with a pump pulse centered at 3.10 eV with a fluence of 700 μJ/cm^2^ (a) and 420 μJ/cm^2^ (b). The red horizontal lines indicate the spectral position of LSPR (2.14 eV) and TSPR (2.37 eV).

Overall, the Δ*A* maps are dominated by two negative signals at the two plasmon energies and a positive signal red-shifted to the LSPR. These spectral features arise from both spectral broadening and the red-shift of the plasmon bands caused by the dielectric constant changes resulting from the electron heating induced by femtosecond photoexcitation. Indeed, several previous works that investigated the TA spectra of noble metal nanostructures revealed that their transient optical response on the picosecond time scale is dominated by the hot electron dynamics.^1,4,14,33^ By exciting in resonance with the SPR, a coherent oscillation of electrons is activated. This oscillation loses coherence very fast (*τ*_dephasing_ ∼ 5–20 fs) through different scattering phenomena, depositing energy into the electron distribution, and creating excited electrons that are spread over different levels in the conduction band. This nonequilibrium distribution of electron–hole pairs can also be reached following the photoexcitation of the interband or intraband transitions of the metal.^4^ In our pump–probe experiment, we are exciting the interband transitions as we pump to the blue of SPR (Figure 1c), which is above the interband onset of about 2.4 eV for Au.^4^ This distribution of excited electrons rapidly thermalizes via electron–electron scattering (*τ*_e__–e_ ∼ 100 fs), losing information about the initial excitation and increasing the electronic temperature *T*_e_. This increase of electron temperature results in a transient broadening, as it accelerates the total dephasing rate, and a transient shift of the SPR.^30−32,35,36,38−40^ Then, electron–phonon coupling (*τ*_e__–ph_ ∼ 1–6 ps) equilibrates the electron and lattice temperature *T*_l_, thereby decreasing *T*_e_ and raising the temperature of the nanoparticle. In addition, if the deposited thermal energy is high enough, the rapid thermal expansion of the metallic nanoparticles induced by electron–phonon coupling can coherently excite its breathing vibrational mode.^34,41,42^ Eventually, the hot particle equilibrates with the environment through phonon–phonon interactions (*τ*_ph__–ph_ ∼ 100–200 ps). The time evolution of electron and lattice temperatures *T*_e_ and *T*_l_ can be described by the well-known two-temperature model (TTM),^4^ which also establishes that, for relatively small induced temperature changes Δ*T*_e_, the evolution of the electron temperature *T*_e_ can be described by the following function^32^
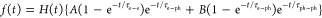
1where *H*(*t*) is the Heaviside function. For small Δ*T*_e_, the time evolution of the electron temperature is directly reflected in the transient absorption, and therefore, equations analogous to eq 1 have often been employed to fit the experimental decay trace measured in TA experiments and to obtain the characteristic time constants.^15,16,23,32^ While this model has yielded in many instances remarkable agreement with the experimental results, permitting a simple, qualitative description of the electron dynamics in many nanostructured metal systems, this approach neglects the energy dependence of the electron relaxation^32^ and does not allow for the distinction of different contributions leading to the above-mentioned broadening and shift following photoexcitation. This is also clearly detectable in our data, where the fit of the Δ*A* data with eq 1 returns different *τ*_e__–ph_ constants at each probe energy with maximum values at the peak positions. This trend becomes more evident at high pump fluences (Figure 3a). Therefore, a more detailed model is needed to correctly account for the experimental behavior. To gain deeper insight into the origin of the observed transient spectra and to disentangle contributions arising from different mechanisms involved in the photoinduced modulation of the absorption at different probe energies, a different fitting model was thus proposed. To directly reveal the broadening and the frequency shift as a function of time and account for the energy dependence, we first fit all of the TA spectra by describing LSPR and TSPR as Voigt profiles:
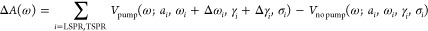
2

**Figure 3:**
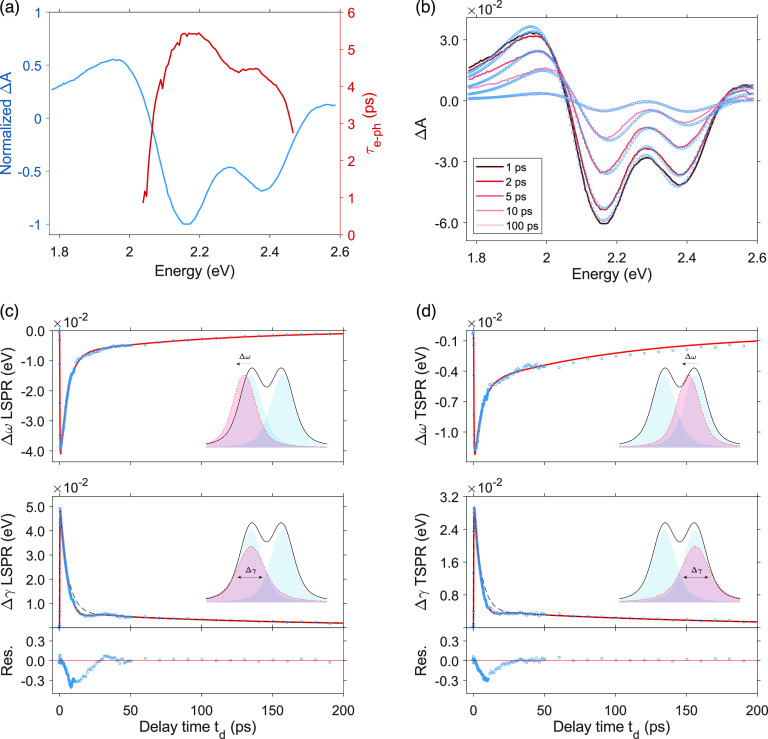
Analysis of the pump–probe data. As an example, here only the results of the analysis performed on the data collected at the highest pump fluence (700 μJ/cm^2^) are reported, but the same procedure was carried out for all of the measurements. (a) Trend of the *τ*_e__–ph_ constants resulting from the direct fitting of Δ*A* with the function in eq 1 at each wavelength (red line). The TA spectrum at a selected value of delay time (1 ps) normalized on its minimum is shown for comparison (blue line). (b) TA spectra at different delay times *t*_d_ (blue dots) and the respective fitting curves obtained by using eq 2 (red lines). (c) Time evolution of Δω (upper panel) and Δγ (central panel) for LSPR. Blue dots, experimental points; red lines, fitting lines obtained with eq 1 for Δω and eq 3 for Δγ. For a better estimation of the short-time behavior, the deconvolution of the system’s response function was performed during each fitting procedure (Supporting Information). The cartoons in the insets provide a pictorial sketch of the associated phenomena (photoinduced spectral shift and photoinduced broadening, respectively). The oscillating residues in the Δγ trace are plotted in the bottom panel. (d) Same as (c) but for TSPR.

The Voigt profile is defined as a convolution of a Lorentzian line shape with a Gaussian frequency distribution, and it is employed to describe the line shape of spectroscopic lines when both homogeneous (dynamic) and inhomogeneous (static) phenomena contribute to the line broadening.^43,44^ The Voigt profile has been selected instead of the more commonly used Gaussian or Lorentzian models (that implies the predominance of inhomogeneous and homogeneous broadening, respectively) based on evidence emerging from 2DES experiments, as described below. In eq 2, Δ*A*(ω) is the TA spectrum as a function of the probe energy ω at a fixed value of delay time *t*_d_, which is modeled as the sum of two contributions, accounting for the LSPR and TSPR, respectively, with the index *i* running over these two contributions. *V*_pump_ (*V*_no pump_) is the Voigt function^43,44^ describing the absorbance of the probe in the presence (absence) of the pump. *a*_*i*_ is the Voigt area, *ω*_*i*_ its central frequency, 2*γ*_*i*_ the full-width-at-half-maximum (FWHM) of the Lorentzian component, and *σ*_*i*_ a parameter associated with the FWHM of the Gaussian component (). Δ*ω*_*i*_ and Δ*γ*_*i*_ quantify the time dependent frequency shift and broadening of the SPR bands promoted by photoexcitation.

The major advantage of this approach is that it allows differentiation of the contributions of broadening and shifting to the nonlinear signal, allowing clear identification of their signatures separately for TSPR and LSPR. This model fitting was globally applied to the TA spectra at each delay time *t*_d_. In order to reduce the number of fitting parameters and reliably extract Δ*γ*_*i*_ and Δ*ω*_*i*_, when possible, the other parameters were estimated or constrained within specific ranges based on other independent measurements, including 2DES, as described in the Supporting Information.

An example of the results of this fitting procedure is reported in Figure 3b, which shows a set of TA spectra extracted at selected values of time delay *t*_d_ and the respective fitting curves obtained by applying eq 2. From these curves, the values of Δ*γ*_*i*_ and Δ*ω*_*i*_ as a function of *t*_d_ were extracted and plotted for LSPR and TSPR in Figure 3c and d, respectively. For both LSPR and TSPR, Δ*ω*_*i*_(*t*_d_) is negative and Δ*γ*_*i*_(*t*_d_) is positive; this means that, in both cases, the SPR absorption at a higher electron temperature is red-shifted and broader than the SPR absorption at ambient temperature *T*_0_. These dynamics were then fit with the function in eq 1. A closer look at the time behavior of the Δ*γ*_*i*_ traces for both LSPR and TSPR reveals the presence of beating residues with a period of 53 ± 5 ps (see the bottom panels of Figure 3c and d). Beatings with a similar period (ranging from 50 to 80 ps depending on the rods’ length) have already been detected in ultrafast measurements of NRs and have been attributed to the breathing mode along the longitudinal axis.^41,42^ A similar attribution can also be assumed here, although longitudinal and transverse breathing modes cannot be considered independent in the case of our low AR rods.^4,45^ These beatings are the results of the rapid thermal expansion of the metallic nanoparticles induced by electron–phonon coupling,^41,42^ and their contribution in the Δ*γ*_*i*_ time traces can be accounted for by introducing the following fitting model
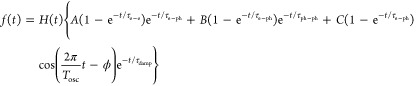
3where *T*_osc_ is the period of the oscillations and *τ*_damp_ their dephasing time.

Figure 4 summarizes the relevant broadening and shift dynamics of LSPR and TSPR as results of the fitting procedure. Let us first focus on the electron–electron scattering rates. Figure 4a and b show the *τ*_e__–e_ values obtained from the fitting of (Δ*γ*_*i*_ vs *t*_d_) and (Δ*ω*_*i*_ vs *t*_d_) traces, respectively, as a function of the pump fluence. The *τ*_e__–e_ values extracted from the photoinduced broadening and photoinduced shift show the same kind of dependence on the pump fluence, while clear differences emerge between the two SPRs. For TSPR, the electron–electron scattering process is slower than that in LSPR (see Figure S5), and the associated time constant *τ*_e__–e_ decreases quadratically with the pump fluence. This quadratic dependence is not clearly detectable for LSPR, but it cannot be fully excluded, considering that kinetics with a time scale in the order of 0.1 ps or shorter are at the limit of the experimental time resolution. This trend is in agreement with the Fermi-liquid theory, stating that the temperature dependence of the electron–electron scattering times can be approximated as .^32,33,46,47^

**Figure 4:**
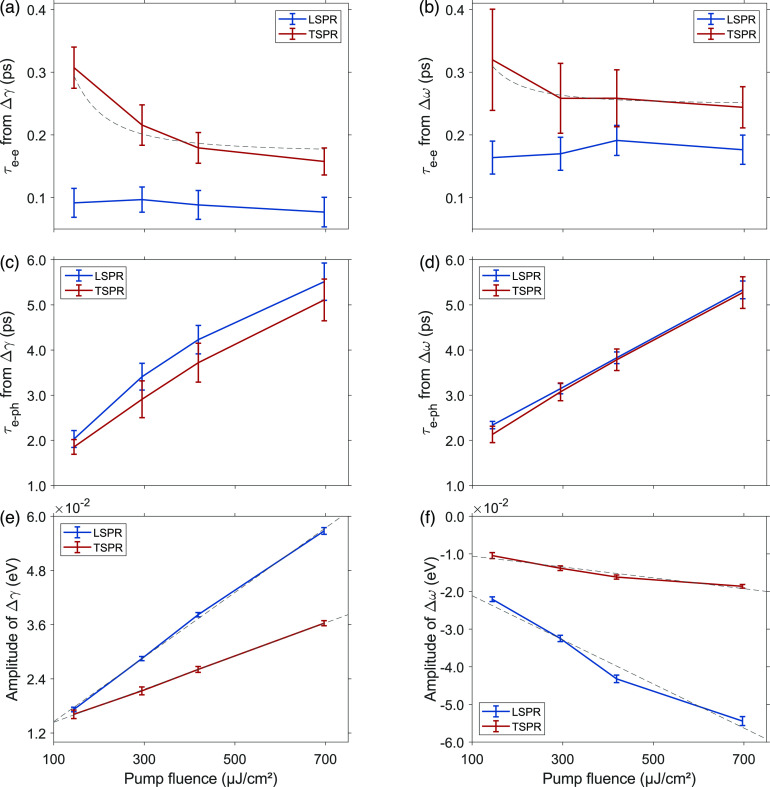
Comparison of the results obtained from the analysis of the pump–probe data at different pump fluences. (a) Electron–electron times obtained from Δ*γ*_*i*_ traces; (b) electron–electron times obtained from Δ*ω*_*i*_ traces; (c) electron–phonon times obtained from Δ*γ*_*i*_ traces; (d) electron–phonon times obtained from *Δω*_*i*_ traces; (e) amplitude of the *Δγ*_*i*_ contribution obtained as the sum of the fitting parameters *A* + *B* (eq 3); (f) amplitude of *Δω*_*i*_ obtained as the sum *A* + *B* (eq 1) at different pump fluences. Error bars represent the 95% confidence interval obtained from fitting data for a single measurement at a specific pump fluence by using eq 1 and eq 3.

Analogously to parts a and b of Figure 4, parts c and d of Figure 4 illustrate the fluence-dependent trends of *τ*_e__–ph_. Figure 4d shows that within the experimental error similar behavior is found for the photoinduced shift of both LSPR and TSPR, with time constants ranging between 2 and 5 ps, increasing with the pump fluence. The fluence trends in Figure 4c are slightly different. The electron–phonon time constants extracted from Δ*ω*_*i*_(*t*_d_) scale linearly with the pump fluence, in agreement with the prediction of the TTM model, while the ones extracted from the Δ*γ*_*i*_ dynamics show a slight nonlinearity at higher fluences and assume consistently greater values for LSPR than for TSPR. An analogous nonlinear behavior of *τ*_e__–ph_ with pump fluence was already noticed in the literature at comparable fluence values, suggesting the establishment of a nonperturbative regime.^30,38,40,48^ While the experimental error does not allow for definitive identification of the saturation behavior, the comparison between the trends reported in Figure 4c and d seems to suggest a higher sensitivity of the photoinduced broadening of the LSPR to the pulse fluence, as discussed also below. The trends reported in Figure 4c and d have then been used to estimate the intrinsic electron–phonon relaxation time *τ*_e__–ph_,_0_. Indeed, in accordance with the TTM, this value can be determined by extrapolating *τ*_e__–ph_ to zero pump fluence (Figure S3), giving an intrinsic electron–phonon relaxation time of *τ*_e__–ph_,_0_ = 1.16 ± 0.36 ps at 25 °C. It is worth noting that this value may be overestimated due to the saturation behavior experimented in this range of pump fluences. In fact, it is important to stress that eq 1 and eq 3 are valid under the approximation of low excitation and may not produce accurate results at higher fluence values. Nonetheless, excluding the last point, the value estimated for *τ*_e__–ph_,_0_ is consistent with prior research findings on gold NRs.^15,29,35,36,48,49^

Finally, the longest time decay *τ*_ph__–ph_ does not show any significant difference between LSPR and TSPR at every pump fluence, and it was estimated to be 145 ± 40 ps. Again, this is a result expected according to the TTM if one assumes that the temperature changes of the solvent can be neglected.^4,48^

Together with the values of the time constants, the amplitudes of the exponential functions in eq 1 and eq 3 also carry significant information. In Figure 4e and 4f we show the total amplitudes obtained from the fitting of (Δ*γ*_*i*_ vs *t*_d_) and (Δ*ω*_*i*_ vs *t*_d_) traces, respectively, calculated as the sum of the pre-exponential factors *A* and *B*, as a function of the pump fluence. As expected, for both of the plasmonic resonances, a linear behavior is found, but also in this case, LSPR and TSPR exhibit significantly different sensitivity to the pump fluence. Indeed, as the pump fluence increases, the contribution of LSPR to both photoinduced broadening and photoinduced shift increases with respect to TSPR. This behavior suggests that the two plasmonic resonances have significantly different nonlinear behaviors.

The last property emerging from the fitting is the beating appearing in the (Δ*γ*_*i*_ vs *t*_d_) traces. The fitting revealed a beating frequency with a period of 53 ± 5 ps. According to previous literature, these beatings are attributed to the particle’s breathing mode activated as the result of the rapid thermal expansion induced by scattering phenomena. The vibrational modes are impulsively excited when the heating is faster than their period. These excited modes generate modulations in the transient absorption traces due to the periodic change in the volume or shape of the particles. Interestingly, differently from previous observations in the literature,^4,48,50−52^ our findings indicate that oscillations exert a significantly greater influence on Δγ as opposed to Δω, where no substantial beating phenomenon was detected. This implies that the microscopic mechanisms regulating the photoactivated nuclear motion do not involve a significant variation of the frequency of SPRs, supporting the hypothesis of a hybrid breathing mode where both axes oscillate with uniform phase and relative intensity, keeping the LSPR’s frequency nearly constant. Furthermore, if there was a change in the AR, one would expect to observe oscillations only at the LSPR’s frequency; however, the same modulation is also observed at the TSPR’s frequency. More likely, the observed beatings can be attributed to a symmetric, periodic modulation of the nanoparticles’ volume, which impacts the dephasing rates of SPRs, consequently influencing the photoinduced broadening Δγ. The phase of the oscillations is estimated to be approximately 93°. This indicates that, during the initial picoseconds, as the particles’ size increases due to the rapid transfer of energy from the electron gas to the lattice,^53^ the homogeneous width of SPRs decreases. This suggests that the modulation of the volume may influence the dephasing rate by altering both the electron density and the contribution of surface scattering (which is inversely proportional to the nanoparticle’s size).^4^

To shed light on the dynamics regulating the first hundreds of femtoseconds and to complement the information at longer time scales obtained by the previously described pump–probe experiments, we analyzed the same NR sample by 2D electronic spectroscopy (2DES). Despite the well-recognized capabilities of the technique to study ultrafast phenomena in nanomaterials,^54^ up to now it has been only rarely employed to explore the ultrafast dynamics of noble-metal nanostructures.^31,47,55^

The output of a 2DES measurement is a three-dimensional matrix in which the nonlinear signal is represented as a function of the excitation frequency (*ω*_exc_), detection frequency (*ω*_det_), and population time *S*^(3)^(*ω*_exc_, *t*_2_, *ω*_det_). Slices of this matrix at fixed values of *t*_2_ lead to the so-called 2D spectra, as shown in Figure 5a. More details about the technique and its experimental implementation can be found in refs (56 and 57). To compare the results of 2DES with the pump–probe ones, it is necessary to remember that the 2D spectrum is typically expressed in electric field units, contrary to TA spectra that are expressed in optical density units. This means that the signs of the 2D and pump–probe spectra are opposite.^58^

**Figure 5:**
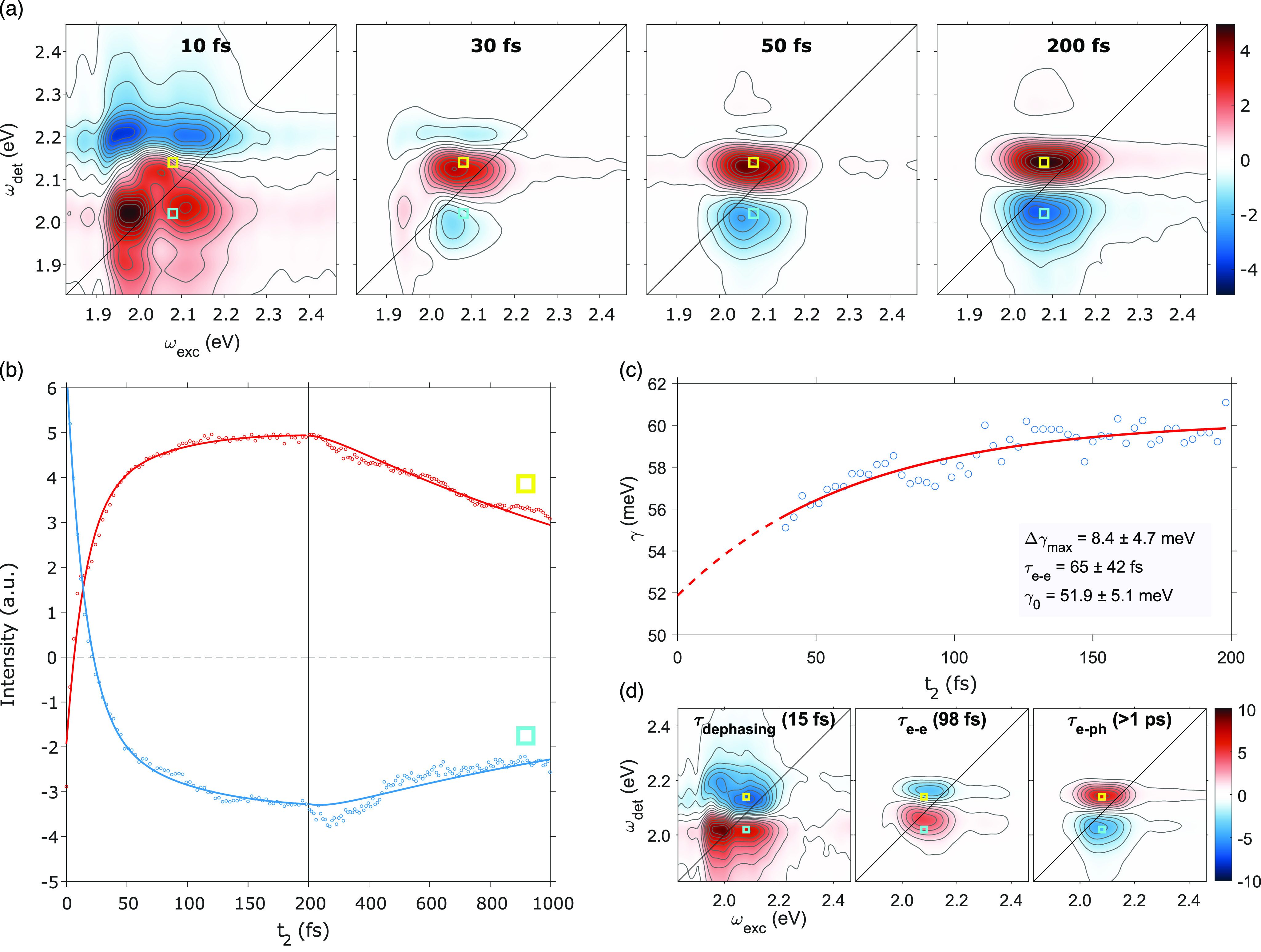
Results of 2DES experiments. (a) Purely absorptive 2DES maps at selected values of population time *t*_2_. (b) Time traces extracted at two relevant coordinates pinpointed by the light blue and yellow squares. (c) Half-width-at-half-maximum of the main peak along the antidiagonal dimension plotted as a function of *t*_2_ (dots = experimental values; solid line = exponential fit). (d) Decay associated spectra (DAS) for the three time constants extracted by the global fitting. The values of the time constants are reported in each plot.

Figure 5a shows the purely absorptive 2DES spectra at selected values of the population time. More maps, including the ones referring to the rephasing and non-rephasing part of the signal, are reported in the Supporting Information (Figure S6). Excluding the first 10 fs, two major contributions to the whole signal can be noticed: a positive (red) and a negative (blue) peak with the same coordinate on the excitation energy axis (*ω*_exc_ = 2.08 eV) and detection frequencies of 2.02 and 2.14 eV, respectively. Considering the spectral profile of the exciting pulse (Figure 1), these signals can mainly be attributed to the nonlinear response of the LSPR, which is resonantly excited in the experiment. Despite the different conditions of photoexcitation, after the plasmon dephasing, the main features in the 2D maps have the same origin as the signal recorded in the TA spectra, i.e., the broadening and red-shift of the plasmon band caused by the photoinduced electron heating. The comparison between the TA spectra and the 2D maps integrated along the excitation frequency, in accordance with the projection slice algorithm,^59^ shows exactly the same behavior (see the Supporting Information, Figure S7). Also the time dependence of the signal is analogous, as shown in Figure 5b where the signal decays extracted at relevant coordinates are shown.

However, the multidimensionality of the 2DES technique and its higher time resolution allow the extraction of more subtle information, which, on the one hand, permits a more solid justification of some of the assumptions conventionally made in the analysis of the TA spectra. On the other hand, it provides complementary information on the phenomena occurring in the ultrashort time window (within the first 100 fs after photoexcitation), which is usually not accessible in TA experiments.

One of the first advantages of multidimensional techniques, recognized since their original development, is the capability of distinguishing between homogeneous and inhomogeneous broadening phenomena.^44,54,59,60^ Over the years, much effort has been spent in the analysis of dynamics and peak shape of the 2D spectra to identify and distinguish these two mechanisms. The simplest way is to compare the diagonal and antidiagonal width of the peak describing a single transition, which gives details of the inhomogeneous and homogeneous line widths, respectively.^59,61^ Another related technique is nodal line slope (NLS) analysis, which exploits the interference of positive and negative value bands in 2D spectra. This analysis has been recently applied to 2D data collected on inhomogeneous ensembles of gold nanorods.^31^

The NLS analysis revealed that, in our data, the NLS is close to zero for the entire time window investigated. According to recent calculations, this corresponds to a situation where the inhomogeneous and homogeneous contributions to the broadening are comparable.^31^ Although the laser pulse can excite only a less heterogeneous subensemble of NRs (Figure 1c), this finding is particularly important because it justifies the choice of a Voigt profile for the fitting of the TA spectra obtained by pump–probe spectroscopy rather than the more usual Gaussian (Lorentzian) model, typically adopted when the inhomogeneous (homogeneous) broadening is prevailing.

We also estimated the time-dependent homogeneous width, by evaluating the antidiagonal line width of the peak appearing in the absolute value 2D maps at each population time *t*_2_. The first 40 fs were excluded because the ultrafast dephasing processes and pulse overlap effects hinder a reliable determination of line widths in such a time window. The results of this analysis, reported in Figure 5c, provide very important findings. First, an estimate of the homogeneous line width *γ*_LSPR_ before the electron scattering phenomena take place could be obtained, by extrapolating the value at *t*_2_ = 0, which resulted in 52 ± 5 meV. At increasing values of *t*_2_, the homogeneous line width progressively increases because, as described before, the initial distribution of excited electrons promoted by photoexcitation rapidly thermalizes via electron–electron scattering. The time dependence of *γ*_LSPR_ thus provides the exact value of the *τ*_e__–e_, estimated by an exponential fitting to be 65 ± 42 fs. Furthermore, the amplitude of the exponential fit (8.5 ± 4.7 meV) is also consistent with the trend depicted in Figure 4e, considering that 2DES measurements were carried out at a laser fluence of about 9 μJ/cm^2^ (an extrapolation from the linear fit in Figure 4e yields a value of 8.0 meV; see Figure S8). It is important to highlight that homogeneous electron dynamics cannot be easily extracted from 1D steady state or pump–probe spectroscopy signals, where the contributions of inhomogeneous and homogeneous line broadenings are strongly intertwined. This analysis thus represents one of the first directly measured pieces of evidence of such mechanisms.

The time evolution of the 2DES maps has also been analyzed through a multiexponential global fitting model.^56^ The fitting procedure resulted in three time constants, 15, 98, and >1000 fs, whose amplitude distribution across the 2D maps can be visualized in terms of decay associated spectra (DAS),^57^ shown in Figure 5d. The second and third time constants, based on their values, the sign and signal distribution in the corresponding DAS, and the comparison with the pump–probe results, can be easily interpreted as *τ*_e__–e_ and *τ*_e__–ph_, respectively. Note that the *τ*_e__–e_ value from the global fitting is in good agreement, within the experimental error, with the value extracted from the line width dynamics.

The shortest time constant has a value comparable to the pulse duration, and the corresponding DAS accounts for an ultrafast change of sign of the signal in the first tens of femtoseconds. In the first tens of femtoseconds after photoexcitation, the photophysical behavior of the NRs is expected to be dominated by the coherent behavior of the plasmon resonance. In fact, within the dephasing time, the system can be described as an ensemble of collective coherent oscillations of electrons coupled with a restoring electromagnetic field. It is also well-known that, when a plasmon resonance is excited by an electric field under resonance conditions, the electron cloud makes a transition between in- and out-of-phase oscillation with respect to the incident wave around the center frequency of the resonance.^6,62,63^ This phase transition might explain the peculiar amplitude distribution associated with the ultrashort time constant, which can thus be related to plasmon dephasing. In addition, the value of 15 fs gives rise to a homogeneous width of about 45 meV (from 2*γcπ* = 1/*τ*_dephasing_),^64^ which is in strong agreement with the value obtained from the antidiagonal’s width analysis. Therefore, although this dynamic behavior is close to the time resolution limit of the measurements and further investigations would be necessary for a final attribution, it is reasonable to interpret the 15 fs time constant as *τ*_dephasing_. Hence, this value was used as an input parameter in the fitting model of eq 2 for pump–probe analysis. This is one of the few direct experimental determinations of the dephasing time of SPR in the literature.^13,65−67^

In conclusion, the pump probe and 2DES have been synergistically employed to clarify the mechanisms underlying the early ultrafast femtosecond processes following photoexcitation in low AR nanorods. An accurate fitting model applied to transient absorption spectra allowed ascertaining that the photoinduced hot electron dynamics for the LSPR and TSPR exhibit significantly different sensitivity to the pump fluence. Moreover, it was possible to clearly differentiate the contributions of broadening and shifting to the nonlinear signal, with the former being more sensitive to the pump fluence and more informative on the coherent displacement of the nuclei due to the thermal expansion following photoexcitation. 2DES experiments completed the description, providing a direct quantification of the plasmon dephasing time and of the homogeneous line width dynamics dominated by electron–electron scattering processes, not achievable with more conventional 1D time-resolved techniques. As a result, we obtain complementary and internally consistent insights into the coherent plasmonic dynamics at the femtosecond time scale and incoherent hot electron dynamics over picosecond time scales. This combined approach, using pump–probe and 2DES techniques, holds crucial significance for comprehending and harnessing the photoresponsive properties of these promising nanomaterials.

## Supporting Information Available

Experimental methods, additional details on fitting models, and additional pump–probe and 2DES data (PDF)
