# Supplementary material for: Coherent and Incoherent Ultrafast Dynamics in Colloidal Gold Nanorods
Source: J Phys Chem Lett. 2024 Jan 3;15(1):339–48. doi: 10.1021/acs.jpclett.3c03226 (PMC10788960; doi:10.1021/acs.jpclett.3c03226)
Supplement: Supplementary file 1 — jz3c03226_si_001.pdf [file jz3c03226_si_001.pdf]

ELECTRONIC SUPPLEMENTARY INFORMATION

Coherent and Incoherent Ultrafast Dynamics in Colloidal Gold Nanorods

Federico Toffoletti[1], *Elisabetta Collini*[1,2]*

[1] Department of Chemical Sciences, University of Padova, via Marzolo 1, 35131 Padova, Italy

[2] Padua Quantum Technologies Research Center, Via Gradenigo 6/A, Padova Italy

*elisabetta.collini@unipd.it

S1. EXPERIMENTAL METHODS

Au NRs CTAB capped synthesis. Synthesis was performed adopting the method proposed by L. Liz-Marzán et al.[1,2] The aspect ratio (AR) of nanorods (NRs) was tuned by gently oxidizing the particle's surface with a solution of Au^{3+}. All the solvents and reactants were obtained from Sigma-Aldrich (Merck KGaA, Darmstadt, Germany) and used as received without further purification.

TEM Measurements and Extinction Spectra. TEM analysis was performed with a jeol 300 PX electron microscope, while extinction spectra were recorded with a Cary 5000 spectrophotometer. Ten images with approximately 300 NRs were analyzed for the dimensional dispersion of the colloidal nanosystems. The results are shown in Figure S1.

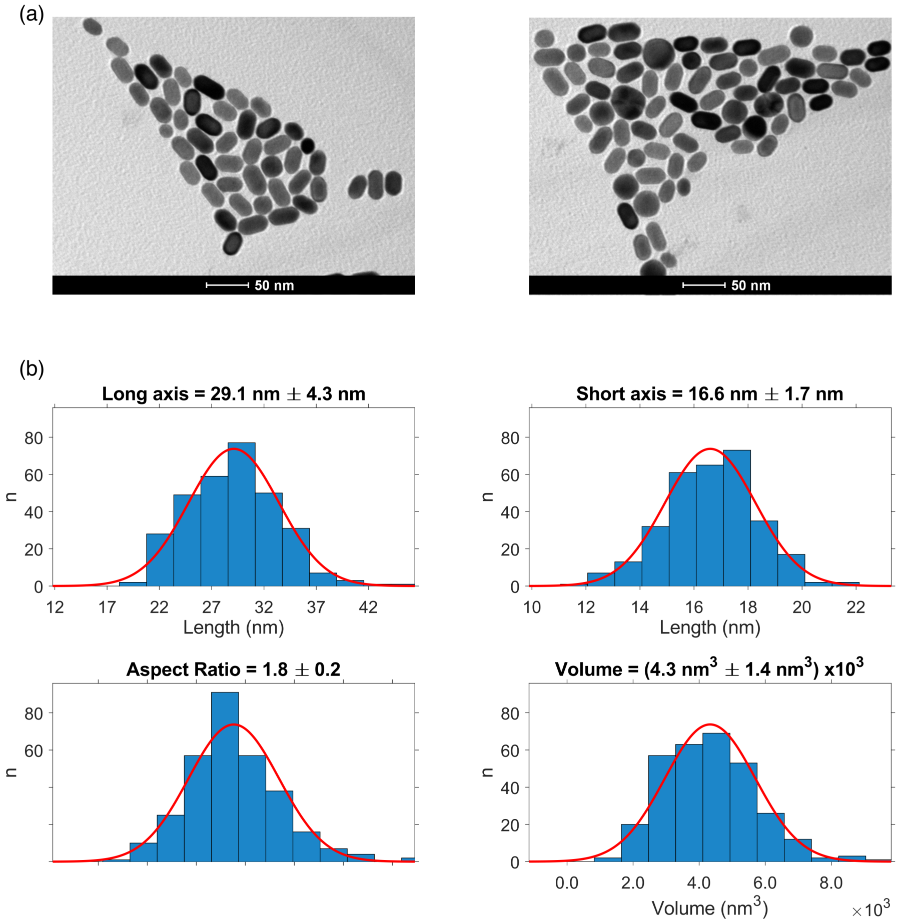

Figure S1. Dimensional characterization. a) TEM images of CTAB capped Au NRs dispersed in water and b) size distributions of the long axis, short axis, AR, and volume, respectively, obtained from the analysis of up to 300 NRs.

Pump-probe Measurements. The pump and the probe beams are generated by an amplified Ti:Sapphire laser (Spitfire, Spectra Physics) seeded by a femtosecond pulsed Ti:Sapphire oscillator (Mai-Tai, Spectra Physics). The laser pulses are emitted at 800 nm, with an energy of 0.8 mJ per pulse, a repetition rate of 1 kHz, and a 180 fs pulse duration. The output laser beam is split by a 4% beam splitter into two paths. The weaker one generates a supercontinuum white light in a thin sapphire plate and is used as the probe. The stronger portion is instead used to generate the pump pulse at 400 nm via second harmonic generation in a BBO thin crystal. The pump fluence is tuned from 100 to 700 μJ/cm^2 using OD filters and its repetition rate is halved to 500Hz through an optical chopper. The collimated pump pulse and the focused probe pulse hit the samples in an overlapping region within the sample. The delay between pump and probe pulses is controlled with a motorized linear stage. The transmitted light is dispersed and directed to a linear CMOS diode array. The quality of the TA signal, i.e., ΔA values (differential absorption), is improved through repeated measurements and averaging (200 measurements were averaged to obtain a sufficient signal-to-noise ratio). The obtained spectra are numerically processed to minimize white light chirping effects by using a homemade Matlab routine. Each measure is repeated at least twice to verify the reproducibility of the phenomena.

A TA spectrum plots the differential absorption $\Delta A(t_d, \omega)$ as a function of the probe energy ω at a fixed value of the time delay t_d after pump excitation:

$$\Delta A(t_d, \omega) = -\log_{10}\left(\frac{I_P(t_d, \omega)}{I_{NP}(\omega)}\right) = A_P(t_d, \omega) - A_{NP}(\omega) \tag{S1}$$

where $I_{NP}(\omega)$ and $I_P(t_d, \omega)$ are the intensity of the signal at probe energy ω without pump excitation and at a time delay t_d after pump excitation, respectively.

2DES Measurements. The experiment is conducted using a 3 KHz Ti:Sapphire Coherent® Libra laser system coupled with a commercial NOPA (Light Conversion® TOPAS White) to generate pulses centered at about 600 nm. A compression of about 11 fs at the sample position is achieved

through a prism compressor coupled with a Fastlite Dazzler pulse shaper for fine adjustment, as determined through frequency-resolved optical gating (FROG) experiments (Figure S2). The 2DES experiment relies on the passively phase stabilized setup, where the laser output is split into four identical phase-stable beams (three exciting beams and a fourth beam further used as Local Oscillator, LO) in a BOX-CARS geometry using a suitably designed 2D grating. Pairs of four CaF_2 wedges modulate time delays between pulses. Delay times t_1 (coherence time between first and second pulse), t_2 (population time between second and third pulse), and t_3 (rephasing time between the third pulse and the emitted signal) are defined. The rephasing and non-rephasing parts of the signal are recorded by acting on the pulse sequence. The coherence time is scanned from 0 to 80 fs with steps of 2 fs for the rephasing experiments and from 0 to 64 fs with steps of 2 fs for the non-rephasing experiments. The population time is scanned from 0 to 200 fs with steps of 3 fs in the first series of experiments and from 0 to 1000 fs with steps of 7.5 fs in the second series. The energy per pulse at the sample position is set to about 8 nJ, which corresponds to a fluence of about 9 μJ/cm^2. The purely absorptive signal is then calculated as the sum of the rephasing and non-rephasing signals. The heterodyne detected third order signal is collected using a double lock-in method. For a more detailed description of the setup and data processing procedures, see ref.[3] Each experiment is repeated at least 4 times to guarantee reproducibility.

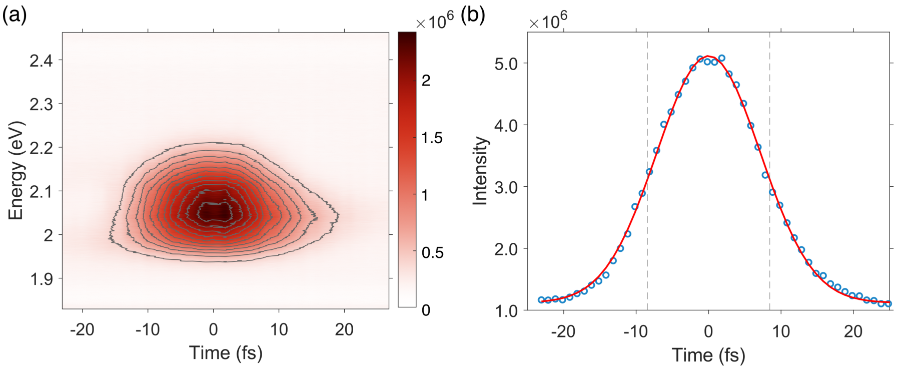

Figure S2. Pulse width characterization. FROG measurement performed in the 2DES setup at the sample position by replacing the sample with dimethyl sulfoxide. The laser pulse duration is estimated to be 11 fs.

S2. ADDITIONAL DETAILS ON FITTING MODELS

Two-temperature model (TTM). Electron and lattice temperatures T_e and T_l are well described by the two-temperature model (TTM) through a set of coupled differential equations:[4,5]

$$\begin{cases} \dfrac{dT_e}{dt} = -g(T_e - T_l)/C_e(T_e) & \text{(S2)} \\ \dfrac{dT_l}{dt} = g(T_e - T_l)/C_l - (T_l - T_0)/\tau_{ph-ph} & \text{(S3)} \end{cases}$$

where $C_e(T_e)$ and C_l are the specific heats of electron and lattice, and g is the characteristic electron-phonon coupling constant. The second term in Eq. S3 represents heat transfer to the surroundings (with temperature T_0) with time constant τ_{ph-ph}. The temperature dependence of $C_e(T_e) = \zeta T_e$ (where ζ = 66 $Jm^{-3}K^{-2}$ for Au) implies that the timescale for electron-phonon coupling depends on the initial electronic temperature. Because of this dependence, accurately measuring the electron-phonon coupling constant is particularly problematic. To overcome this problem, a series of different measurements at different pump laser fluences can be used. At low excitation levels, the relaxation time τ_{e-ph} is given by $\zeta(T_0 + \Delta T)/g$, where ΔT is the temperature increase caused by the pump laser. Therefore, the characteristic electron-phonon time $\tau_{e-ph,0} = \zeta T_0/g$ can be obtained by extrapolating the result at zero fluence, as shown in Figure S3. The linear relationship found between τ_{e-ph} and the pump fluence confirms the fulfillment of the small ΔT_e regime (low excitation level) and the validity of the predictions based on the TTM model.

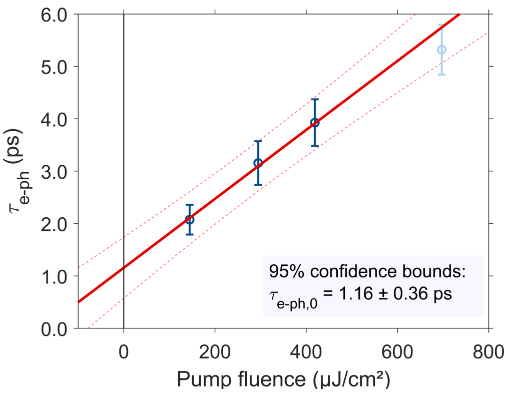

Figure S3. Characteristic electron-phonon time determination. Linear fit of all the τ_{e-ph} values obtained from $\Delta\gamma_i$ and $\Delta\omega_i$ traces as a function of the pump fluence. The points are obtained as the mean of the four values at that specific fluence, and the error bar is estimated through their standard deviations. The extrapolation at zero fluences gives a characteristic electron-phonon time of about 1.16 picoseconds.

Initial guess of fitting parameters. The broadening and red shifting dynamics were obtained by fitting the TA spectra with Eq. 2. In order to reduce the number of fitting parameters and reliably extract $\Delta\gamma_i$ and $\Delta\omega_i$, the other parameters were estimated or constrained within specific ranges based on other measurements. The energies ω_{LSPR}, and ω_{TSPR} are easily extracted from the extinction spectrum. For what concerns the other parameters relative to LSPR, σ_{LSPR} was determined through the analysis of the AR dispersion from TEM images. The trend of LSPR frequencies as a function of the AR found in our synthesis is displayed in Figure S4. Using this, we could easily translate the AR standard deviation ($\sigma_{AR} = 0.2$) into a corresponding standard deviation for the LSPR frequencies, yielding an estimate of 78.4 meV. The homogeneous broadening γ_{LSPR} was estimated experimentally (i) from the dephasing time measured in 2DES and (ii) from the antidiagonal width of the main 2DES signal. The two values are in agreement and provide a value of about 48 meV. On the other hand, direct measurements of σ_{TSPR} and γ_{TSPR} are not easily accessible. However, we expect that the inhomogeneous width of TSPR should be lower compared to that of LSPR since the energy of TSPR changes minimally with the NR size. Tentative fittings with slightly different values of both σ_i (within a ± 10% interval) did not show significant differences. Furthermore, considering that the TSPR has

energy closer to the interband transitions (2.4 eV for Au),[4] it is expected to exhibit a larger homogeneous width due to interband scattering effects.[4,6] In light of these considerations, the parameters a_i , γ_i, and σ_i for *i*=TSPR were selected to achieve the best fit with the data for each TA spectra while still maintaining the constraints mentioned before. The select parameters are summarized in Table S1.

Table S1. Fitting parameters.

a_{LSPR} (eV)	a_{TSPR} (eV)	γ_{LSPR} (eV)	γ_{TSPR} (eV)	σ_{LSPR} (eV)	σ_{TSPR} (eV)	ω_{LSPR} (eV)	ω_{TSPR} (eV)
0.067	0.058	0.045	0.056	0.082	0.061	2.14	2.37

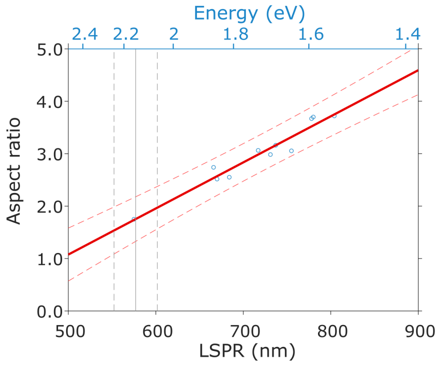

Figure S4. Gold Nanorods LSPR vs AR. Linear relationship between the LSPR wavelength and the AR obtained from different Au NRs synthesized in our lab. From the slope of this linear trend, we converted the dispersion in the AR of the investigated sample ($\sigma_{AR} = 0.2$) to the inhomogeneous dispersion of LSPR energy (σ_{LSPR}=78 meV).

Additional details on the fitting functions. Here the description of the Voigt function employed for the fitting of transient absorption (TA) spectra:

$$Voigt: \qquad V(\omega; a, \omega_0, \gamma, \sigma) = a \int_{-\infty}^{\infty} G(\omega'; 0, \sigma)\, L(\omega - \omega'; \omega_0, \gamma)\, d\omega'$$

$$Gaussian: \qquad G(\omega; \omega_0, \sigma) = \frac{1}{\sigma\sqrt{2\pi}} e^{\frac{-(\omega-\omega_0)^2}{2\sigma^2}} \quad ; \quad FWHM_G = 2\sigma\sqrt{2ln2}$$

$$Lorentzian: \quad L(\omega; \omega_0, \gamma) = \frac{1}{\pi} \frac{\gamma}{(\omega - \omega_0)^2 + \gamma^2} \quad ; \quad FWHM_L = 2\gamma$$

where a is the Voigt's area, ω_0 is the frequency center, γ the Lorentzian's width and σ the Gaussian's width.

For a better estimation of the short-time behavior, the $\Delta\gamma(t)$ and $\Delta\omega(t)$ dynamics were fitted using a function that incorporates the convolution of the system's response function with the instrumental response function (IRF), which is approximated as a Gaussian pulse with a duration of 150 fs (σ_{pulse} of 63.7 fs):

$$f(t) = \int_{-\infty}^{\infty} RDD(t)\, G\big(t - t'; 0, \sigma_{pulse}\big)\, dt' \qquad \text{(S4)}$$

where RDD is the function described in Eq.1 or Eq.3 of the main text.

The 2DES data analysis was performed through a complex multi-exponential global fitting model which has been proposed in our group.[7] The fitting function $f = \sum_{n=1}^{N} a_n e^{i\phi_n} e^{-t_2/\tau_n} e^{-i\omega_n t_2}$ was used to fit simultaneously all the signal decay at each coordinate of the 2DES maps, and it allows simultaneous access to both the oscillating ($\omega_n \neq 0$) and non-oscillating ($\omega_n = 0$) components, each one associated with a specific kinetic constant τ_n.

S3. ADDITIONAL DATA

Table S2. Results from the analysis of pump-probe data.

$\tau_{e-ph,0}$ (ps)	T_{osc} (ps)	τ_{damp} (ps)	τ_{ph-ph} (ps)	Δγ LSPR (meV/nJ)	Δω LSPR (meV/nJ)	Δγ TSPR (meV/nJ)	Δω TSPR (meV/nJ)
1.16 ± 0.36 [a]	53.4 ± 5.4 [b]	6.1 ± 2.2 [b]	138 ± 41 [b]	72 ± 8 [a]	59 ± 31 [a]	36.7 ± 1.7 [a]	14 ± 11 [a]

[a] Error represent the 95% confidence interval obtained from linear fitting of data collected at various pump fluences.

[b] Error of the parameters that remain constant with respect to the pump fluence, determined by the standard deviation of multiple measurements.

Table S3. Results from the analysis of 2DES data.

Global fitting				Antidiagonal HWHM		
$\tau_{dephasing}$ (fs)	→ γ_0 (meV)	τ_{e-e} (fs)	τ_{e-ph} (fs)	γ_0 (meV)	τ_{e-e} (fs)	Δγ LSPR (meV)
14.8 ± 1.5 [a]	44.5 ± 4.6 [a]	98 ± 26 [a]	1603 ± 40 [a]	51.9 ± 5.1 [a]	65 ± 42 [a]	8.4 ± 4.7 [a]

[a] Error of the parameters determined by the standard deviations of four different measurements at the same laser fluence (9 μJ/cm^2).

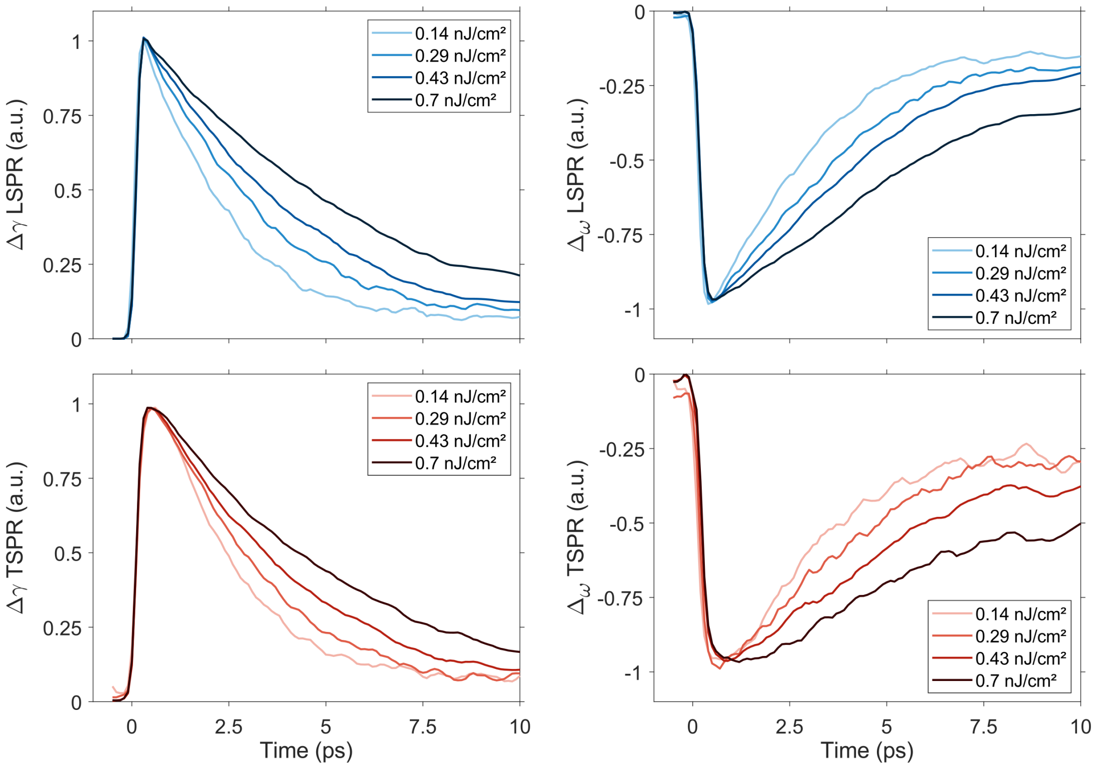

Figure S5. $\Delta\gamma_i$ and $\Delta\omega_i$ dynamics. Normalized time traces of broadening and red shift of longitudinal (blue) and transverse (red) SPRs at different pump fluences in the first 10 ps. Both the electron-electron and the electron-phonon dependence on pump fluence are clearly visible. It is worth highlighting the distinct dynamic behavior of TSPR and LSPR in the initial 1-2 picoseconds. This difference is then reflected in longer electron-electron times at the TSPR frequencies, as discussed in the main text.

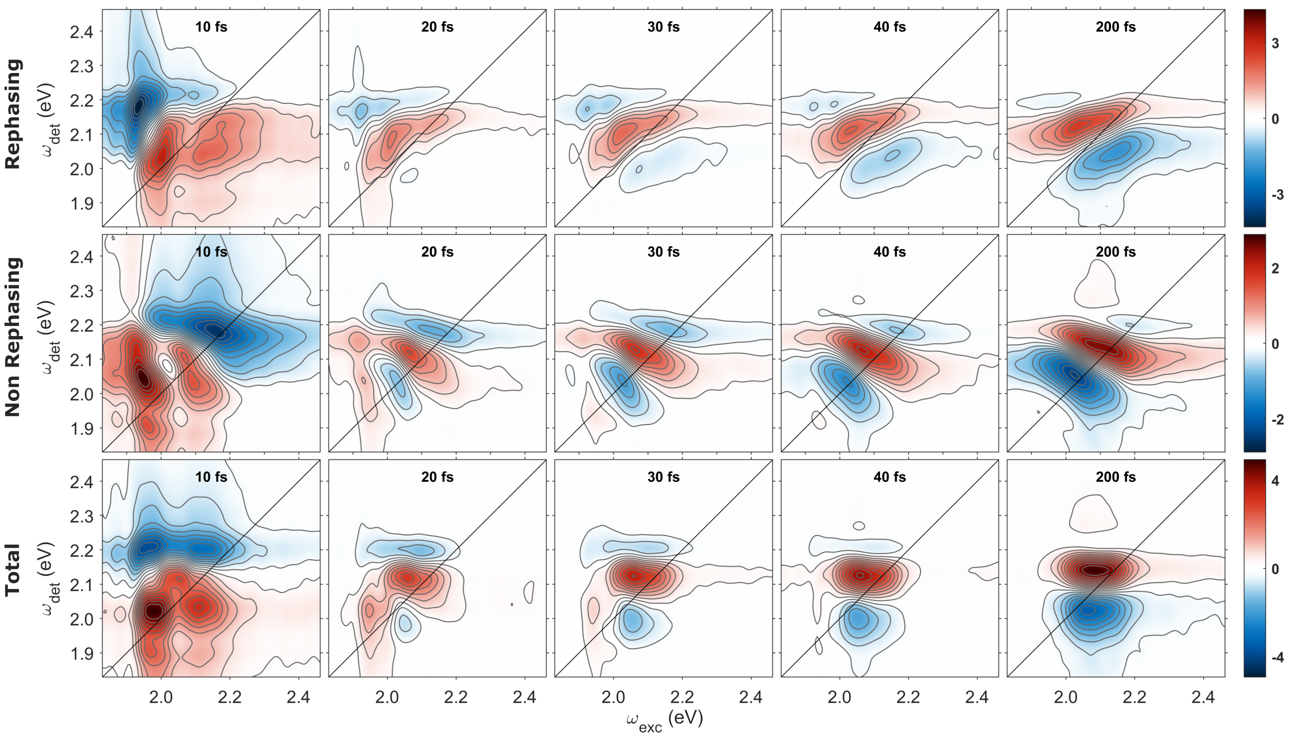

Figure S6. 2DES Maps. Rephasing, Non Rephasing and Total (purely absorptive) 2DES maps collected at 10, 20, 30, 40 and 200 fs.

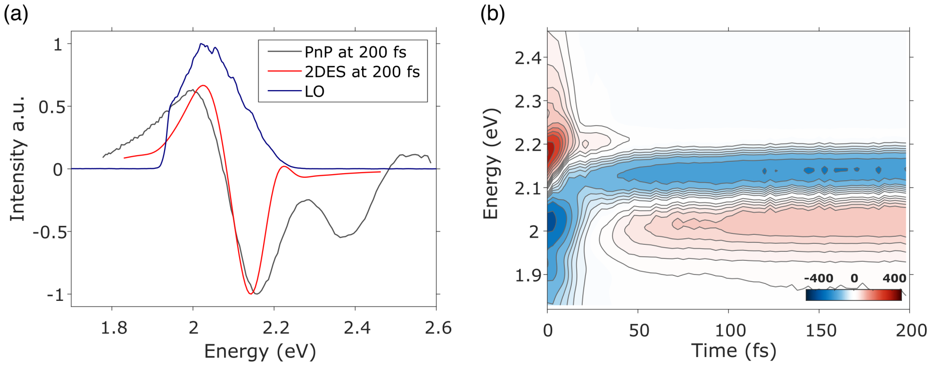

Figure S7. 2DES and pump-probe. The phasing of the 2DES data was performed through the comparison with pump-probe data (projection slice theorem). In panel (a) the TA spectrum at 0.2 ps is compared with the 2DES map integrated along the ω_{det} axis, acquired at 200 fs. Panel (b) displays a bi-dimensional plot representing the differential absorption derived from the 2DES measurement. These images confirm the perfect agreement between 2DES and pump-probe experiments.

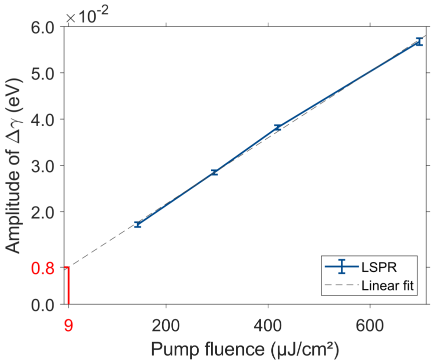

Figure S8. Amplitude of $\Delta\gamma$. From the linear fit of the amplitude of $\Delta\gamma$ as a function of pump fluence, we can extract a value of 0.008 eV at a pump fluence of 9 μJ/cm². This value is in strong agreement with that obtained through the analysis of 2DES data.

REFERENCES

(1) Rodríguez-Fernández, J.; Pérez-Juste, J.; Mulvaney, P.; Liz-Marzán, L. M. Spatially-Directed Oxidation of Gold Nanoparticles by Au(III)−CTAB Complexes. *J Phys. Chem. B* **2005**, *109* (30), 14257–14261.

(2) Scarabelli, L.; Sánchez-Iglesias, A.; Pérez-Juste, J.; Liz-Marzán, L. M. A "Tips and Tricks" Practical Guide to the Synthesis of Gold Nanorods. *J. Phys. Chem. Lett.* **2015**, *6* (21), 4270–4279.

(3) Bolzonello, L.; Volpato, A.; Meneghin, E.; Collini, E. Versatile Setup for High-Quality Rephasing, Non-Rephasing, and Double Quantum 2D Electronic Spectroscopy. *J. Opt. Soc. Am. B* **2017**, *34* (6), 1223.

(4) Hartland, G. V. Optical Studies of Dynamics in Noble Metal Nanostructures. *Chem. Rev.* **2011**, *111* (6), 3858–3887.

(5) Hartland, G. V. Coherent Excitation of Vibrational Modes in Metallic Nanoparticles. *Annu. Rev. Phys. Chem.* **2006**, *57* (1), 403–430.

(6) Sun, C.-K.; Vallée, F.; Acioli, L. H.; Ippen, E. P.; Fujimoto, J. G. Femtosecond-Tunable Measurement of Electron Thermalization in Gold. *Phys. Rev. B* **1994**, *50* (20), 15337–15348.

(7) Volpato, A.; Bolzonello, L.; Meneghin, E.; Collini, E. Global Analysis of Coherence and Population Dynamics in 2D Electronic Spectroscopy. *Opt. Express* **2016**, *24* (21), 24773.
